# Municipal Government: Its True Sphere and Duties

**Published:** 1856-04

**Authors:** 


### JOURNAL OF PUBLIC HEALTH.

MARCH 1856.

### MUNICIPAL GOVERNMENT; ITS TRUE SPHERE AND DUTIES.*

In modern London life, few events have caused a greater social sensation than the changes in municipal government, introduced by the new Act for the better local management of the metropolis. Our citizens are one and all on the *qui vive*, and are exchanging ideas on the probable results of the measure, with that generous flux of speech which is common to all small-talk conversations, where the speaker knows that the opinions he hears will not modify the course of events one jot, and that those which he gives, having the same weight, will not compromise him in the least degree, even should they turn out fallacious in the course of the ensuing day.

It is not our intention, on the present occasion, to enter into an inquiry regarding the probable results of the new metropolitan measure, or the strength of its political basis. Time will be the best and only faithful critic in these regards. We intend to take a more general view, and to indicate what the duties of municipal government in this country ought to embrace, irrespective for the moment of the regulations or restrictions of existing laws. We are emboldened in this task, by having before us a philosophical though brief treatise on "Municipal Government", from the pen of Mr. Roberton, of Manchester. Of this gentleman's valued labours we shall avail ourselves freely in succeeding paragraphs.

Vestry labours of the past.

As an introductory word, we cannot do better than refer at once to an observation made by the author above named, to the effect, that the whole duties of vestries, up to the present time, have seemed

* Suggestions for the Improvement of Municipal Government in Populous and Manufacturing Towns. By John Roberton, Surgeon. Manchester: 1855. The Metropolis Local Management Act. London: 1855.

to consist in the correction of nuisances, lighting, cleansing, paving, sewering, rounding off corners, widening streets, opening new thoroughfares, supplying water, and keeping up an effective police. These duties have of course implied others of a dependent kind, such as the infliction and collection of rates. But, necessary and important as they are, they embody scarce a fractional part of the wider social government now urgently demanded. If the new Act for the metropolis serves only to support with a little more efficiency the limited duties just narrated, it will fail signally, and had better not have been brought before the legislature at all.

The high and low rate.

The true usefulness of municipal government will depend ultimately, in a great measure, upon the breadth of view with which its representatives are able to grasp the various questions connected with social economy and monetary expenditure. As yet, the hero of the municipal assembly has been the man who could most eloquently declaim on the abstract question of the high or low rate; while he who has been venturesome enough to descant on the best and widest uses to which a rate, either high or low, might be applied, has been left to run for his reputation. To rectify this error is reform number one; for to strain at a farthing, and swallow a nugget, is an act fatal at the onset to social advancement.

Physical & moral conditions of districts, a point of inquiry.

The construction of a history of the physical and moral conditions incident to the district over which it presides, is a primary duty of each local parliament. It is true that, amongst honourable councillors, there may be found one here and there who, from long residence in his locality, has vague ideas regarding some of its leading advantages and defects. But such knowledge is exceptional, and of no practical utility; for what is local is of the general, and scraps of unbound information are but play toys for an idle wind of argument, which may make a terrible noise, but of which no one knows whence it cometh nor whither it goeth.

Viewed, however, in a general and philosophic sense, social facts of all kinds present in themselves no greater difficulties than do those of natural science; to which, in truth, they belong. The social world has its gradations and its epochs; its first, second, and third series. It has its revolutions, diurnal, annual, and illimitable. It may be put in the mental alembic, and reduced to certain elementary conditions. It has its simples, its usefuls, and its poisons. In fine, the man of general science may find in social studies the choicest scientific pursuits, subject in the strictest sense to forms of experimental research, and to the rules of the inductive philosophy. These facts lie at the bottom of social reform; and social science can never progress until our amateur legislators learn them as a language, the A B C of which but few as yet know.

Education.

The best means for introducing into each locality a liberal, practical, and attainable system of education for the poorer and neglected portion of the population, constitute another important subject for the earnest consideration of all municipal boards. If they wait until the parliament of the nation makes a law on this point, independent of external pressure, they may possibly wait long, with half their removable social evils, which education only can affect, thick upon them. For this ultimatum they ought not to wait. Neither should trust any longer be placed on private exertions in this direction; for private enterprise, however well intentioned it may be, is subject to fluctuations, and often dies out after a short and brilliant career. We require permanent oases of education in the barren desert of English ignorance. Permanent ragged schools, permanent day schools, permanent evening schools for working men and women—one evening school to every twelfth gin-shop, for a rough example. These are desiderata which are required for each district; modified, of course, according to the character of the district itself. They can never be supplied, except by wise local legislation, or be permanently supported but by the cheapest in the long run of all taxation—an educational rate.

Protection of neglected infants.

We would presume to throw out a hint (following in the footsteps of Mr. Roberton) regarding the establishment, under municipal authority, of houses for the protection of young and neglected children during the labour hours of the day. In Paris an instructive experiment in this department of social science has been, indeed, for some years in operation.. In the day nurseries of the French capital, from two to three thousand babes are housed, tended, and, if old enough, taught, while the mother is out following her industrial occupations. Some improvements might, it is said, be made in these establishments, but this does not remove the fact that such institutions are greatly needed; for the system, as a whole, is based on most sound and humane principles, and is even more applicable to England than to France. In London, some twenty thousand infants, at least, who might be thus cared for, are left each working day of life to the careless protection of an elder sister, the charity of a neighbour, or, far worse, to the narcotic mercies of a compound poison invented by one Godfrey, who had better never have been born. What say you, then, Mr. Municipal Councillor, to a few day nurseries on this side the Channel? If you have a child of your own, bethink you, on the strength of paternal love, whether an imitation on your part of this Parisian fashion would not only be—as such imitations usually are—highly popular, but calculated also to bring honour to your name, and greater happiness to your own fireside.

Play - grounds and gymnasia.

In every parish, large or small, there ought to be established gymnasia and play-grounds for children. Cooped up from morning to night in narrow, unlighted, unventilated courts, like celery plants banked from the light for the sheer purpose of being bleached, our younger denizens of large towns have no knowledge of the glowing sensation which one swift current of oxygenated blood communicates to the body. Hence they rise up incapacitated, physically and mentally—victims of struma, victims of sloth, victims of ignorance. But, as physical and mental vigour are one and the same, and as pure air and exercise are first principles in the natural code of health, so should they be provided for in any and every local administrative act relating to the health of the community.

Protection of outcast women.

The protection of outcast females, and the institution of reformatory dwellings for their benefit, are other subjects which should engage the attention of local legislative bodies, if rightly constituted. Labours in this direction would, in fact, be sanitary as well as social. Indeed, in their absence, sanitation can make but indifferent way, medical officers of health though there may be in hosts, able and active. For, mark you, honourable lords and councillors all, in Parliament, in vestry, or elsewhere, by these outcasts there is kept up the greatest, most universal, and persistent epidemic with which mortals are verily cursed; an epidemic which, in its way, we assure you, induces indirectly more real physical suffering, more misery, more diseases, more mortality, than all the other epidemics in total, cholera inclusive. It is at the same time a preventible epidemic, being the offspring of a social and removable evil. The key to the removal of this quiet and wholesale plague is presented in one sentence—find or patronize some useful and paying occupation, which shall supply the mere necessaries of life to the miserable creatures who propagate it. Degraded as a class, ranking as the lowest victims of sensual loathsomeness, they themselves would gladly as the American slave see the day of their emancipation at hand. We pray you, then, forward it; and let not the Saxon race degenerate, as it is now fast doing, into a dense herd of consumptives, coughing up their lives, dying in their prime, and leaving to another, and yet another generation, the germs of their own premature decay.

Female occupations.

The general question of female occupations is one which requires to be carefully surveyed and discussed, irrespective of the reformatory results which might and would follow from the inquiry. So long as we commit the absurd error of talking of the physical and mental feebleness of woman, and yet see her worked like a dray-horse, we are erring grievously. In a correct organisation, there could be no anomalies of this character. They are unnatural, perverse, foolish. Is shirt making at fourpence a day always to remain the highest and most profitable occupation for the poor daughters of Eve? Are they never to be liberated from the troubles of sixteen, nay, even twenty-four hours per diem, in the brute exercise of factory labour? Is the temptation of selling their beauty to crime, to save their bodies from starvation, never to be removed? We hope better. But in these, as in other matters, the spirit of regenerative action must wake up in the municipal bodies, to whom the way will be open everywhere, if the will shall become good. The establishment of model lodging houses for the special advantage of industrious females, is, we believe, within the jurisdiction of municipal authority even now. It is impossible to say how gracious a boon an effort in this single direction would become, or how thankfully it would be received.

Encouragement of art and industry.

In the work of social advancement for men and women of all classes, the liberal encouragement of art and industry should never be neglected. Emulation is a certain inducement to progress; and there is no reason whatever, except apathy and narrow statesmanship, against the introduction into every large parish of an industrial exhibition or bazaar, which might be as permanent as the church, and where year by year, or even day by day, the intellectual talent of the locality might be exhibited to the mental advantage of all, and the profit of the industrial exhibitor.

Workhouse administration.

Workhouse management, again, requires from municipal authority stringent and immediate reform. Workhouses, from the fact that the occupants are under direct control, should be model institutions. Dr. Sieveking's suggestion for the education of nurses might well be put into active operation; and the children here reared, if the learning supplied to them were practical, industrial, and artistic, might be turned out to become the most useful and intelligent servants of the community.

Medical charities.

Under an enlightened system of municipal administration, the medical charities of the different districts of every large town would be advantageously supervised. We would not suggest for a moment that the supporters of these charities should be shorn of that administrative power which their philanthropy has purchased. But the facts are notorious, that these institutions are often grossly mismanaged, because they are every body's property, and are therefore managed by nobody; while the medical staffs are unrewarded for their unremitting labours. With the governors of such charities, then, the municipal bodies might co-operate; they might depute one of their members as a representative in each charity; they might check selfish or spendthrift management in one case, they might give a helping hand in another—they might supervise all. The same observations apply to asylums for the insane, and to houses of refuge for the destitute supported by benevolent exertions.

Beggary & crime.

The stricter prohibition of street begging, and the lightening of the labours of the magistrate by removing the inducements to crime, are points on which we shall not dwell, because the suggestions already made would, if carried out, remove many of these evils as a matter of necessity. Some temporary alleviations are, however, required urgently; amongst which night refuges for the absolutely destitute, in parishes where workhouse accommodation is limited, are perhaps the most important, as means of raising human kind from one of the lowest depths of callous degradation.

Sanitary measures.

In regard to the great question of sanitary supervision, many of the duties of the municipal councils are embodied in that which we have already written. The removal of nuisances, the constant observation of disease, the supply of pure water, the building of baths and washhouses, the prevention of overcrowding, the securing of ventilation, the supervision of articles intended for human consumption—these duties are all understood by legislators, and will soon, it is naturally expected, be generally performed. Two hints only shall we offer in extension. First, that in considering the diseases of human flesh and blood, those of the inferior animals ought not to be neglected, seeing that the laws of disease are universal, and that the intercommunication of disease between man and his lower earth mates is of frequent occurrence. Secondly, that the effects of various occupations on health and life form an inquiry, in this country, open to serious and immediate consideration. In France, as we have elsewhere pointed out, the governing bodies have reviewed this subject in all its bearings, and have legislated wisely upon it.

There are points connected with street accidents, the possibility of making the ordinary policeman a sanitary inspector under proper medical superintendence, and some others, to which allusion might well be made here. But, with another word, we must conclude.

A municipal reformer wanted.

In order that any new reform may be carried out in municipal government, one sterling, honest, comprehensive soul is waited for to lead the way. Is it possible that there is a want here? Can it be that, in all the municipal councils of England, there is not one man, dead to fear, but alive to truth, who, in the face of all opposition, misapprehension, prejudice, and selfishness, will proclaim the fact, that municipal government is as yet in a stage of almost helpless infancy, and who will shadow out that strong manhood to which it is expected to attain? For the honour of our country, we trust this is not the fact, and that the time will furnish the man. If it be urged that municipal governments have not the power to carry out many of the most needed social reforms, our answer is, let them then get the power. Active, business-like, wise, and liberal local governments, would do more in the way of reforming our general parliaments than aught else that could be conceived; for the people would soon detect who were their wisest rulers, and the vestry might become the training school for the leader in the state.

We have done. Some reader, perchance, as his eye falls on these last lines, may cry *Cui bono?* may lay down the book with a smile, and dub us utopian speculators. Ah, well! the reproof is tolerable. We have earnestly, honestly written the truth—not the whole truth, for that were impossible, but a fractional part at least. For the rest—

"Our faith is large in time,
And that which fits it to some perfect end."

